# Confirmation of a low HER2 positivity rate of breast carcinomas - limitations of immunohistochemistry and in situ hybridization

**DOI:** 10.1186/1746-1596-5-50

**Published:** 2010-07-29

**Authors:** Ulrich F Vogel

**Affiliations:** 1 Institute of Pathology, University Hospital, Eberhard-Karls-University, Liebermeisterstrasse 8, 72076 Tuebingen, Germany

## Abstract

**Background:**

Accurate assessment of the human epidermal growth factor receptor 2 (HER2) of invasive breast cancer is essential to treatment decisions since the advent of targeted therapy with the humanized monoclonal antibody trastuzumab and the dual tyrosine kinase inhibitor lapatinib. In the literature, the percentage of HER2-overexpressed/amplified breast carcinomas range from 3% to 30%. The routinely assigned low rate of 9% of HER2-overexpressed breast carcinomas alarmed one of our gynecologists who requested to confirm our HER2 test results.

**Methods:**

A small study of 83 patients with breast carcinoma was designed to reexamine the routinely assessed HER2 status using immunohistochemistry and fluorescence in situ hybridization.

**Results:**

The low rate of 9% of HER2-overexpressed/amplified breast tumors (DIN1C-3, invasive carcinoma) could be confirmed. However, FISH revealed two false positive cases and one false negative case. Moreover a case with an equivocal result in FISH was detected.

**Conclusion:**

The HER2 positivity rate may be as low as 9%. The novel ASCO/CAP criteria for assessing immunohistochemical results in HER 2 testing reduce the false positive rate of HER2. First-line testing with immunohistochemistry may obscure false positive and false negative test results. In heterogeneous carcinomas even fluorescence in situ hybridization may not succeed in a correct evaluation of HER2.

## Background

The assessment of human epidermal growth factor receptor 2 (HER2) in invasive breast cancer is mandatory for treatment decisions since the advent of targeted therapy with the recombinant humanized IgG monoclonal antibody trastuzumab (Herceptin^®^, Genentech, Inc., South San Francisco, CA, USA; Hoffmann-La Roche Ltd., Basel, Switzerland) and the small molecule dual HER1/HER2 tyrosine kinase inhibitor lapatinib (Tykerb, GlaxoSmithKline, Philadelphia, PA, USA) [1]. The trastuzumab antibody binds to the extracellular domain of HER2, a transmembrane tyrosine kinase receptor, and results in growth inhibition and apoptosis of tumor cells overexpressing HER2 [2,3]. This overexpression of the HER2 protein correlates with mRNA levels and the amplification of the HER2 gene, allowing immunohistochemical and in situ hybridization (ISH) assays for determining the HER2 status of a cancer cell [4]. Scores have been defined to evaluate the immunohistochemical tests semiquantitatively (0-3+; Clinical trial assay Score, HercepTest-Score) and ISH assays quantitatively (e.g. Vysis-Ratio), which were slightly modified recently by the American Society of Clinical Oncology and the College of American Pathologists [5]. However, the best method to assess the HER2 status remains controversial [5]. According to Mass et al., only patients with HER2 gene amplification likely benefit from therapy with trastuzumab, indicating that the preferred method for selecting patients for antibody therapy is the assessment of HER2 amplification by fluorescence in situ hybridization (FISH) [6]. Presumably due to cost-effectiveness, however, screening of all newly diagnosed breast carcinomas is mostly performed by immunohistochemistry (IHC). In contrast to ISH techniques like FISH, IHC is prone to false negative and false positive results due to inappropriate tissue handling, and may even lead to false positive results due to staining artifacts [7,8]. The rate of HER2-overexpressed/amplified breast carcinomas varies in the literature from 3% to 30%, but is mostly given as 18-20% [4,5,9,10]. Therefore, this researcher was alarmed by one of our gynecologists, who argued that our HER2-positivity rate of breast carcinomas was too low compared to published data, probably due to incorrect test results. Therefore, this researcher designed a small study to reevaluate the routinely determined HER2 status using IHC, manual dual-color FISH (PathVysion, Abbott molecular) and a technique to construct a paraffin tissue microarray (PTMA) using paraffinized needle biopsy specimens (PNBSs).

## Methods

A total of 96 consecutive needle biopsy specimens from 88 different epithelial breast tumors, 8 non-invasive (ductal intraepithelial neoplasia (DIN) 1C-3) and 80 invasive, from 83 female patients were obtained from the slide and paraffin block archive of the Institute of Pathology of the University of Tuebingen, punched from January 2007 to May 2007 in a center for breast carcinoma of a referring hospital. DINs were also included in the PTMAs because of the need to assess hormone receptor status (data not shown). Following puncture tissue had been immediately fixed in neutral buffered, alcohol stabilized formalin 4,5% for between 6 and 18 hours, and routinely processed and paraffin-embedded. Diagnoses were made, and hormone and HER2 status evaluated.

A PTMA was constructed using PNBSs as described elsewhere [11]. In short, a conventional bare paraffin block was predrilled [12]. Of 96 PNBSs 84 PNBSs contained an appropriate quantity of tumor, were punched out of the paraffin blocks (donor blocks), placed in an ordinary steel mold on a hot plate at 65°C to free the PNBSs from the surrounding paraffin, picked up with a spiky instrument, and manually transferred and inserted into the holes of the predrilled PTMA (number of holes: 187; diameter of the holes: 1.4 mm; distance of the holes: 0.3 mm; depth of the holes: 5 mm). The filled PTMA was fully melted using a double-sided adhesive tape and an x-ray film [13,14]. All further staining was performed on 3-μm PTMA sections mounted on coated slides and baked overnight at 37°C.

The rabbit polyclonal anti-HER2 antibody (Clone A0485, DakoCytomation, Hamburg, Germany), which is also used in the Dako HercepTest approved by the US Food and Drug Administration (FDA) for the assessment of the expression of the HER2 protein in breast carcinomas, was applied in a standardized in-house laboratory technique which was validated by the German proficiency testing program for HER2 [4,15].

Using the PathVysion kit (Abbott Molecular, Abbott Laboratories, Des Plaines, IL, USA), FISH was performed according to the guidelines of the manufacturer.

Evaluation of the HER2 status was performed according to manufacturer's instructions (Abbott molecular, Dako) and the ASCO/CAP guidelines, i.e. in IHC a four graded system (0-3+) and in ISH a three graded system (non amplified, equivocal, amplified) were utilized [5] (Table 1). To determine the HER2 positivity rate using FISH, the FDA-approved cut-off ratio (HER2 signals/chromosome 17 signals) of 2.0 was used (Table 1).

**Table 1:** Scoring systems for HER2

HER2 IHC Scoring		
**Score**	**Pre-ASCO/CAP **Scoring Interpretation/Staining pattern	**ASCO/CAP** Scoring Interpretation/Staining pattern
**0**	Negative/No staining Staining in < 10% of tumor cells	Negative/No staining Staining in <10% of tumor cells
**1+**	Negative/Faint/barely perceptible **in**complete membrane staining in >10% of tumor cells	Negative/Faint/barely perceptible **in**complete membrane staining in >10% of tumor cells
**2+**	Weakly positive/Weak to moderate complete membrane staining in >10% of tumor cells	**Equivocal**/Weak to moderate complete membrane staining in >10% of tumor cells
**3+**	Strongly positive/Strong complete membrane staining in >**10**% of tumor cells	Positive/Strong complete membrane staining in >**30**% of tumor cells
**HER2 FISH Scoring**		

**Pre-ASCO/CAP Scoring** Interpretation/Ratio (HER2/CEP17)		**ASCO/CAP Scoring** Interpretation/Ratio (HER2/CEP17)
Negative/<2.0		Negative/< 1.8
		**Equivocal**/1.8-2.2
Positive/>= 2.0		Positive/> 2.2

HER2 human epidermal growth factor receptor 2; IHC immunohistochemistry; FISH fluorescence in situ hybridization; ASCO American Society of Clinical Oncology; CAP College of American Pathologists; CEP17 chromosome enumeration probe 17 (probe for the centromer of chromosome 17)

## Results

### Routinely assessed HER2 status by IHC

**Figure 1:**
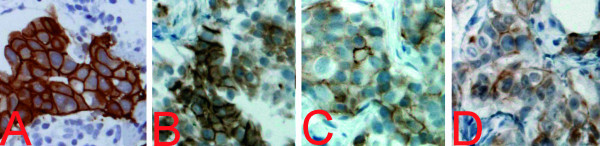
**Immunohistochemical HER2 status of selected cases**. A Correctly identified immunohistochemical HER2 status scored as 3+ (positive) which proved to be amplified by FISH. B False positive immunohistochemical HER2 status originally scored as 3+ which proved to be not amplified by FISH (Table 2, case 3). C False negative immunohistochemical HER2 status scored as 1+ which proved to be amplified by FISH (Table 2, case 4). D Immunohistochemical HER2 status scored as 1+ which proved to be equivocal by FISH (Table 2, case 5). A-D Dako clone A0485. ×20.

Of a total of 88 breast tumors (DIN1C-3, invasive carcinomas) consecutively diagnosed in the first half of 2007, 73 tumors (83%) revealed no expression of HER2 (score 0) by our routine immunohistochemical assay, evaluated according to the pre-ASCO/CAP guidelines. Five tumors were scored as 1+ (6%), one tumor as 2+ (1%; low overexpression), and eight tumors as 3+ (9%; high overexpression; DIN: n = 2; invasive ductal carcinoma: n = 6) (Figure 1A). In one case (1%; DIN1C) the HER2 status could not be determined due to loss of tumor material in the deeper stained sections. Therefore, 87 breast tumors were successfully scored for the immunohistochemical HER2 status.

### HER2 status using FISH

The HER2 status of these 87 tumors was reassessed by FISH on either the PTMA, or on whole mount sections of the PNBSs if the tumor could not be inserted successfully into the PTMAs. Using FISH, eight tumors displayed amplification of the HER2 gene (DIN1C-3: n = 2; invasive ductal carcinoma: n = 6), one tumor had an equivocal score (HER2/chromosome 17 ratios: 1.6-2,1) and one tumor showed polysomy 17 (HER2/chromosome 17 ratio: 1.1). 77 tumors displayed a diploid, non-amplified genomic status. Thus, the HER2 positivity rate, defined as HER2 amplified tumors/all tumors, was 9% (8/87; DIN1C-3, invasive carcinoma) or 8% on invasive carcinomas alone(6/80).

### Comparison of the routinely assessed immunohistochemical HER2 status and FISH

Comparison of the routinely assessed immunohistochemical HER2 status and FISH revealed that two invasive carcinomas diagnosed immunohistochemically as highly overexpressed (score 3+) did not show amplification of the HER2 gene by FISH, with the conclusion that these are false positives (Table 2; cases 2 and 3; Figure 1B). Moreover, one tumor showed amplification by FISH, with about 5-8 HER2 gene copies/tumor cell nucleus, although it was originally diagnosed immunohistochemically as not overexpressed (score 0/1+). This has to be regarded as a false negative (Table 2, case 4; Figure 1C). One tumor, which scored as 2+ by IHC, was proven to be amplified on initial testing with FISH and confirmed in the study setting (Table 2, case 1). Case 5, which proved to be equivocal by FISH, was initially scored as 0/1+ by IHC (Table 2, case 5; Figure 1D). One tumor with an immunohistochemical score of 0/1+ showed a polysomy 17 by FISH (HER2/chromosome 17 ratio: 1,1; Table 2, case 6).

**Table 2:** HER2 status of selected cases.

	Routine PNBSs IHC	Study PTMA IHC	Study PTMA FISH
Case 1	2+	2+	A
Case 2	3+	ND	NA
Case 3	3+	0/1+	NA
Case 4	0/1+	0/1+	A
Case 5	0/1+	0/1+	E
Case 6	0/1+	ND	PS

A amplified; E equivocal; FISH fluorescence in situ hybridization; IHC immunohistochemistry; NA not amplified; ND not done; PNBSs paraffinized needle biopsy specimens; PS polysomy chromosome 17; PTMA paraffin tissue microarray.

### Comparison of the routinely assessed immunohistochemical HER2 status with the immunohistochemical results in the study setting

No discrepancy was found between the routine and the study assessment of the immunohistochemical HER2 status by this researcher using the ASCO/CAP criteria; the false negative tumor described above was also not detected in the study setting (Table 2, case 4).

## Discussion

Alarmed by one of our gynecologists who argued that our HER2 positivity rate of 9% of breast tumors (DIN1C-3, invasive carcinoma) was too low in contrast to most of the data presented in the literature (18-20%) [5,9,10], this researcher designed a study to reassess the routinely determined immunohistochemical HER2 status and to compare the immunohistochemical results with the gold standard dual color FISH, using PNBSs. The PTMA technique was successfully applied to PNBSs. The FISH test confirmed the low HER2 positivity rate of 9%. A larger, ongoing trial in this laboratory with about 1000 breast carcinomas reveals a preliminary positivity rate of 12%. In another study performed 5 years ago in this laboratory, the HER2 positivity rate was 11% (17/151 cases) (unpublished data). The discrepancy of this data to the published HER2 positivity rates of up to 30% is likely due to a different subset of patients, e.g. patients with high-risk early-stage breast cancer and patients with metastatic disease, in the older published studies [5]. Newer studies approximate the same HER2 positivity rate as our findings. Yaziji describes 17% of breast cancers being amplified in a study comprising 2913 cases [9]. Bilous et al. reported a HER2 3+ positivity rate of 12% in a large study with 1536 breast carcinomas, and Chia et al. published a rate of 10.2% of HER2 positive breast cancers in their study cohort of 4444 invasive breast cancers [16,17]. Therefore, the incidence of HER2 positive breast carcinomas was cut into half in contrast to the early reports in the 1990's.

Although the routinely detected immunohistochemical HER2 positivity rate was confirmed by FISH, one false negative and two false positive cases of routine IHC resulted. The terms "false negative" and "false positive" are based on the assumption that FISH testing with a cut-off HER2/chromosome 17 ratio of 2.0 is the best method of correctly identifying patients who are likely to respond to trastuzumab therapy. The two false positive cases (Table 2, cases 2 and 3; Figure 1B) were scored as 3+ by the first investigators due to a 10% strong complete membranous expression of HER2 on the tumor cells. The pre-ASCO/CAP guidelines, which were still applied in the first months of 2007 in our institute, determined the cut-off percentage for an immunohistochemical 3+ score to be 10% of tumor cells with a strong complete membranous expression of HER2 (Table 1). If the ASCO/CAP guidelines with a cut-off percentage of 30% had been applied, the two false positive cases would not have been reported since a FISH test would have been initiated. The two false positive tumors would have been scored as 1+ by this researcher; however the decision, whether 10% or less of the tumor cells show a complete membrane staining for HER2, appears to be sometimes very difficult. 25% (2/8) of false positive (not-amplified) tumors is high; however, Layfield et al. describe in their study that 22 cases out of 79 immunohistochemically scored 3+ tumors were also nonamplified by FISH (28%) [18].

The number of false negative cases in this study (1/77 (0/1+); 1%) (Table 2, case 4; Figure 1C) corresponds to the published data. According to Sauter et al., 2-8% of the carcinomas immunohistochemically scored as 0/1+ show amplification of the HER2 gene, and may be suitable for trastuzumab therapy [8].

One case (Table 2; case 5; Figure 1D) proved to be equivocal according to the ASCO/CAP guidelines with a HER2/chromosome 17 ratio ranging from 1,6 to 2,1 in different evaluations. A correct scoring of this tumor seems to be impossible due to a somewhat heterogenous tumor cell population with 2 to 7 HER2 signals per tumor cell nucleus. Furthermore, the possibility of split signals in FISH and the examination of cut nuclei in paraffinized sections in contrast to cytological examinations may contribute to this problem, and occasionally makes it difficult to determine with certainty the number of HER2 gene copies per tumor cell nucleus. The prevalence and importance of tumor heterogeneity is controversial within the literature. In contrast to Press et al., who described only 0.4% of heterogeneous tumors, Pertschuk found up to 54% [19,20]. Brunelli et al. reported on 13% of HER2 amplified tumors with focally non-amplified tumor areas predominantly in low grade amplified tumors [21]. Tubbs et al. described approximately 5% heterogeneous tumors, which is in the range of our rate of 3%, as in two other cases in this study the HER2 ratio varied between averages of 1.6 and 1.7 [22]. The large variation in the literature may be explained by different definitions of heterogeneity. Can heterogeneity be already diagnosed if some tumor cells show different numbers of the HER2 gene, or is heterogeneity reserved for tumors with a substantial portion of different cells e.g. 50%? It is likely that there is a wide range of tumors, especially in the low level amplification setting as described by Brunelli et al., which display markedly different numbers in amplified tumor cells, leading to different ratios due to inhomogeneous distribution. Case 5 should be reported as "equivocal" to the clinicians according to the ASCO/CAP guidelines. However, this researcher has the experience that the clinical colleagues are not content with the term "equivocal" because they desire clear-cut statements concerning the therapy. Probably, this case may be classified as "not amplified and not suited for therapy with trastuzumab" because the ratio was mostly below the cut-off of 2.0, according to FDA approved guidelines, and did not overexpress in IHC tests performed.

In case 6 (Table 2), FISH revealed a polysomy 17 with up to 8 HER2 gene and chromosome 17 signals per tumor nucleus. The HER2/chromosome 17 ratio was 1.1, indicating a nonamplified status. Whether according to Shah et al. a high polysomal 17-associated HER2 gene copy number is a significant contributing factor in HER2 protein overexpression in unamplified invasive breast carcinomas, and whether those cases should be eligible candidates for treatment with trastuzumab, may be still a matter of debate [23]. According to Bartlett et al. and Watters et al., an increase in the HER2 gene due to an increased chromosome 17 copy does not qualify for amplification [24,25]. In addition to a ratio of 1.1, trastuzumab therapy would not have been indicated in our case due to an immunohistochemically determined 1+ score, indicating no overexpression of the HER2 gene.

Although this study comprises only a small number of cases, the results reflect the ongoing dispute on the favorable technique to prove the HER2 status of cancer cells. Should IHC remain the first-line test and FISH restricted to the immunohistochemical 2+ cases as it is done in most laboratories? There may be several equally valid answers to this question which depend on the goal we want to achieve by the testing, and what degree of uncertainty we will accept.

The variable error rate associated with IHC analysis of paraffin-embedded tissues is well-recognized in the literature to show both false-negative and false-positive results [26]. IHC is influenced by autolysis, fixation, and epitope retrieval, and semi-quantitative scoring is prone to inter- and intraobserver variability. Neither proficiency testing nor on-slide controls may totally prevent inaccurate results. If a certain level of false negative cases is acceptable, then testing may continue. If the goal is solely to avoid false positive immunohistochemical testing in order to prevent unnecessary myocardial toxicity and avoid high treatment cost, then all HER2 positive cases (scores 2+/3+) should be retested with ISH before trastuzumab treatment, as already performed in Belgium and Australia [27,28]. As described by Dendukuri et al, the strategy with the best cost-effectiveness ratio involves screening all newly diagnosed cases of breast cancer with IHC and confirming scores of 2+ or 3+ with FISH testing [29]. Also, Cuadros et Villegas are in favour of testing 2+ and 3+ cases by ISH because of IHC-ISH discordance rates among cases with IHC 2+ and IHC 3+ [30]. However, if the goal is to reduce false positive and false negative rates to less than 1%, then ISH techniques like FISH have to be utilized as the first-line test. Especially FISH has proven to be the best method of correctly identifying patients with metastatic breast cancer who are likely to respond to trastuzumab therapy [8]. Only rare cases respond to trastuzumab therapy without proof of HER2 gene amplification. FISH is both relatively independent of tissue fixation and highly reproducible between laboratories. Failure of FISH testing is reported to be 1-5%, with about 2% on average [6,8]. This high FISH success rate is correlated with analysis of DNA, which is the most stable macromolecule being evaluated, in contrast to analysis of RNA and protein, as performed in IHC [8]. Despite high analysis costs for FISH testing, in contrast to IHC, Elkin et al. showed that it is more cost-effective to use FISH alone as the first line assay, rather than using FISH to confirm only weakly positive results or using the immunohistochemical HercepTest alone [31]. A more cost-effective way to determine HER2 status of all tumors by ISH may be the use of PTMAs, either in the form of PNBSs or tissue cores of the resection or mastectomy specimens [26]. To prevent the time-consuming retrieval of adequate tumor paraffin blocks from the archives, PNBSs or tissue cores could be punched at the time of routine handling and inserted in a consecutively filled PTMA or a storing board over a certain period of time, as published earlier [32]. Missing of heterogeneous tumor components may be a disadvantage of the PTMA technique, due to sampling errors. However, this may also happen when the HER2 status is detected on the small tumor volumes of the PNBSs. As demonstrated by case 5 of our small study even FISH may not result in an exact evaluation of the HER2 status if different Vysis-Ratios are determined in different countings.

Despite our extensive efforts to prevent false positive and negative testing, HER2 testing remains inefficient, as only 12-34% of 3+ amplified breast carcinomas respond to trastuzumab monotherapy [3]. So, until there is agreement on the need for a gene array-based assay to examine the signalling and effector pathways of the HER2 receptor initially in each case, we will continue testing with the gold standard FISH and other methods like IHC which has to be called imperfect as already pointed out by Dietel et al. [33].

## Conclusions

The HER2 positivity rate of breast tumors (DIN1C-3, invasive carcinoma) may be as low as 9%. IHC as first-line testing for HER2 may result in false positive and false negative results. The novel ASCO/CAP criteria for the immunohistochemical evaluation of the HER2 status may prevent some false positive results. The most robust method for studying HER2 status is ISH; however, correct ISH scoring may be impossible and misleading in heterogeneous tumors with only a slight increase in the HER2 copy number. If the paradigm of screening for HER2 status by IHC remains unchanged, an ISH should be performed before treatment with trastuzumab to detect false positive cases. Moreover, all immunohistochemically screened cases should be retested using the PTMA technology if false positive and false negative cases are to be prevented. Until gene array-based assays to evaluate the HER2 receptor pathway are available, the gold standard for assessment of HER2 status in the future will remain ISH, e.g. FISH.

## Competing interests

The author declares that they have no competing interests.

## Authors' contributions

The author designed, performed, wrote and evaluated the study by himself.
